# Editorial: Pathogen-host interaction mechanism in animal models, volume II

**DOI:** 10.3389/fcimb.2026.1858081

**Published:** 2026-05-12

**Authors:** Lihong Xue, Haojie Wang, Qiang Zhang, Xiao yuan Wei, Changyou Xia, He Zhang

**Affiliations:** 1 State Key Laboratory of Animal Disease Control and Prevention, Harbin Veterinary Research Institute, Chinese Academy of Agricultural Sciences, Harbin, China; 2 College of Veterinary Medicine, Huazhong Agricultural University, Wuhan, China; 3 University of South Florida Tampa, Tampa, FL, United States

**Keywords:** animal, mechanism, pathogen, vaccine, veterinary

## Introduction

Pathogen infection is one of the most serious factors threatening the development of the animal husbandry industry. At present, vaccination remains the primary method for controlling animal diseases. Although vaccines against various animal diseases have been developed successively, many still suffer from drawbacks such as poor safety and low protective efficacy. Therefore, the development of novel immunization strategies has become a major challenge for researchers in the field of animal disease prevention and control. A deep understanding of the molecular mechanisms underlying pathogen-host interactions will provide a solid scientific foundation for disease prevention and control, as well as key theoretical support for the development of antiviral drugs, vaccines, and therapeutic antibodies. In the field of animal disease diagnosis, although various new diagnostic technologies have been applied in clinical testing in recent years, challenges such as insufficient sensitivity, low accuracy, and slow detection speed remain. Hence, the continued exploration of new diagnostic technologies is still necessary. Whether based on pathogen detection or antibody detection, these approaches hold promise for providing faster, more accurate, and more convenient tools for the clinical diagnosis of disease outbreaks. This Research Topic, published in Frontiers in Cellular and Infection Microbiology, represents an important exploration of the above-mentioned issues.

## Immune mechanisms of pathogen-host interactions and the development of antiviral drugs

Interactions between pathogens and host cells play a central role in infection, immune evasion, and pathogenicity. Several studies in this Research Topic have revealed the complexity and multidimensional nature of these interactions. One study demonstrated that SIRT7 deficiency significantly inhibits the inflammatory response induced by *Glaesserella parasuis* (GPS) by affecting signaling pathways such as PI3K-Akt, TNF, and cGMP-PKG, indicating that SIRT7 is a key regulator of GPS infection-related inflammation and a potential intervention target (Zheng et al.). Another study identified, for the first time, CD147 as an auxiliary receptor for avian influenza virus, which mediates viral adsorption on the cell surface through direct interaction with the hemagglutinin(HA)protein (Wang et al.).In the context of antiviral drug development, reverse genetics technology serves as a core tool for studying viral pathogenic mechanisms and screening antiviral drugs. In this Research Topic, focusing on porcine reproductive and respiratory syndrome virus(PRRSV), researchers constructed a BAC-based reverse genetics platform using a highly pathogenic PRRSV strain. They successfully inserted the enhanced green fluorescent protein(EGFP)gene into the intergenic region between ORF4 and ORF5a, generating a recombinant virus that stably expresses the reporter gene while maintaining replication kinetics comparable to those of the parental virus. Using this visualization platform, the research team successfully screened four candidate compounds with anti-PRRSV activity, providing an efficient tool for the development of anti-PRRSV drugs (Liu et al.). Another study rapidly constructed reverse genetics systems for two mild coronaviruses, HCoV-OC43 and HCoV-229E, using yeast-based TAR cloning technology, including infectious clones stably expressing reporter genes and replicon systems based on NanoLuc luciferase activity (Zhang et al.). Furthermore, another study successfully established two stably polarized BHK-21 cell models exhibiting either highly antiviral or highly proviral phenotypes against foot-and-mouth disease virus through heavy ion mutagenesis. Multi-omics analysis revealed that this phenotypic difference stems from systemic remodeling of the host transcriptional network, particularly the reprogramming of the immune-metabolism-cell cycle axis, offering powerful tools for the development of high-yield vaccine substrates and antiviral strategies (Song et al.). Several potential antiviral drugs were also identified in this Research Topic. One study demonstrated that fenofibrate, as a PPARαagonist, significantly enhances the defense of bovine mammary epithelial cells against *Mycoplasma bovis* through a dual mechanism: promoting autophagy (upregulating LC3B and LAMP2, promoting TFEB/TFE3 nuclear translocation) and regulating cholesterol homeostasis (inhibiting HMGCR and SREBF2, upregulating ACAT1). This provides a novel strategy for the prevention and control of cell wall-deficient pathogens (Xu et al.). Natural products also showed immunomodulatory potential. Garcinone C was confirmed to significantly inhibit pseudorabies virus(PRV)replication by suppressing the EGF/PI3K/Akt signaling axis, thereby alleviating histopathological damage and improving the survival rate of mice, offering a candidate drug to address vaccine failure caused by the continuous evolution of PRV (Lv et al.).

## Development of novel diagnostic technologies for animal disease

Accurate and rapid pathogen detection is the first line of defense in epidemic prevention and control. This Research Topic reports on several innovative diagnostic technologies. Addressing the global challenge of porcine reproductive and respiratory syndrome virus(PRRSV), researchers developed a duplex real-time RT-PCR method targeting the ORF6 gene, which enables simultaneous detection and differentiation of PRRSV-1 and PRRSV-2. This method demonstrates superior sensitivity and specificity compared to conventional methods, effectively reducing the risk of false negatives caused by viral mutations (Tian et al.).In addition, in this Research Topic, one study classified the novel PRRSV-1 strain ZJ01 as a distinct new sublineage through whole-genome sequencing and phylogenetic analysis, and evaluated its pathogenicity (Ren et al.). For the detection of *Pasteurella multocida* and Salmonella in yaks, a duplex real-time PCR method was established, with a detection limit as low as 10–100 copies. The method was successfully applied to 226 yak samples from the Qinghai–Tibet Plateau, revealing the infection prevalence of these two pathogens in the region (Pan et al.).In addition to real-time PCR technologies, isothermal amplification techniques, which do not rely on precision temperature control equipment, show great potential for point-of-care testing. One study successfully developed a duplex LAMP-LFD(loop-mediated isothermal amplification coupled with lateral flow dipstick)method for the simultaneous detection of Streptococcus suis and Glaesserella parasuis. This method allows rapid completion under constant temperature conditions, with results visible to the naked eye and easy operation, making it particularly suitable for resource-limited basic laboratories and livestock farms. The establishment of these novel diagnostic methods provides powerful tools for early warning and precise prevention and control of animal diseases (Wang et al.). This Research Topic also highlights the application prospects of novel biological products in the prevention and control of animal diseases. A review systematically elaborates on the unique advantages of Nanobodies in the diagnosis and treatment of animal infectious diseases, including their small molecular weight, high stability, ease of production, and engineering adaptability. The review further envisions the broad prospects of Nanobodies in the development of rapid diagnostic kits and novel therapeutic agents (Wang et al.).

## Development of novel vaccines against animal diseases

Finally, this Research Topic emphasizes the importance of vaccine research in the prevention and control of animal diseases. Researchers successfully expressed the E2 protein of atypical porcine pestivirus(APPV)using an E.coli prokaryotic expression system and demonstrated that, when formulated with the ISA 201VG adjuvant, it induced high-titer antibodies in pigs and provided complete protection, laying the foundation for the commercialization of an APPV subunit vaccine (Huang et al.). Targeting the SARS-CoV-2 Omicron variant, researchers constructed a ferritin nanoparticle vaccine (JN.1-4S1158) displaying optimized spike proteins. This vaccine induced broad-spectrum neutralizing antibody responses in mouse models and provided effective protection against multiple Omicron subvariants(BA.5, XBB, JN.1), demonstrating significant potential as a universal coronavirus vaccine. Furthermore, for *Mycoplasma synoviae*, researchers screened a multivalent subunit vaccine(MSPB, Ppht, Cfba, EF-G)through genomics and epitope prediction, achieving an immune protection rate of 90–100% (Feng et al.). Another study in this Research Topic explored the immunomodulatory effects of Rubia-processed *Terminalia chebula* polysaccharides, confirming their ability to effectively alleviate cyclophosphamide-induced immunosuppression in poultry, thereby providing a new approach for developing green and efficient immunopotentiators (Wang et al.). Finally, addressing the risk of zoonotic diseases, a review focuses on the potential association between bovine leukemia virus(BLV)and human breast cancer, emphasizing the public health significance of developing preventive vaccines to block its transmission (Yu et al.).

## Conclusion

We anticipate that in-depth analysis of the immune evasion mechanisms of pathogens and the revelation of the multidimensional regulatory networks of host immunity will provide more effective targets for precision immune intervention. In terms of detection technologies, there is an urgent need to enhance the performance and application scenarios of multiplex detection systems to achieve efficient diagnosis and timely control during disease outbreaks. Regarding vaccine and antibody development, efforts should focus on creating safer and more effective products, as well as advancing research on personalized and precision therapeutic strategies. The studies collected in this Research Topic systematically present the latest breakthroughs in pathogen-host interactions, novel diagnostic methods, and the development of vaccines and antiviral drugs. These findings provide a theoretical foundation and technical support for the creation of next-generation vaccines and disease surveillance, and are of great significance for interrupting the transmission chains of animal infectious diseases, reducing economic losses, and promoting the sustainable development of animal husbandry.

